# Improving patient experience through co-designed patient decision aids in glaucoma

**DOI:** 10.1038/s41433-025-04113-5

**Published:** 2025-11-26

**Authors:** Christin Henein, Jit Kai Tan, Caroline Kilduff, Rashmi G. Mathew

**Affiliations:** 1 National Institute for Health Research Biomedical Research Centre for Ophthalmology Moorfields Eye Hospital and UCL Institute of Ophthalmology, London, United Kingdom; 2 UCL Institute of Ophthalmology, London, United Kingdom

**Keywords:** Health services, Quality of life

Glaucoma management is inherently complex, encompassing a range of treatment options. Shared decision-making (SDM) has become increasingly recognised as a critical component of patient-centred glaucoma care. Patient decision aids (PDAs) play an essential role in supporting SDM by enabling patients to engage in their treatment through the provision of clear, evidence-based information which patients can understand [1]. Distinction between PDAs and information leaflets is important, as PDAs provide information to patients about their choices regarding their disease, which can include observation. In this study, the PDAs were co-produced through a multidisciplinary approach involving students, clinicians, and patients, ensuring content was clinically robust.

Pre-defined glaucoma subtypes and corresponding treatment options were selected for PDA development in accordance with NICE guidelines [2]. Landmark clinical trials relevant to each condition were identified to inform the content, with key statistics extracted to generate explanatory figures and diagrams. Initial versions of the PDAs were co-produced by MSc students as part of a formative assessment, under the supervision of module leads (RGM, CH).

Questions were prepared in advance for the patient focus group (see Supplementary File 1). Insights from the focus group were recorded and analysed using thematic analysis, with revisions implemented based on emergent themes. This is summarized in Fig. 1.

**Fig. 1:**
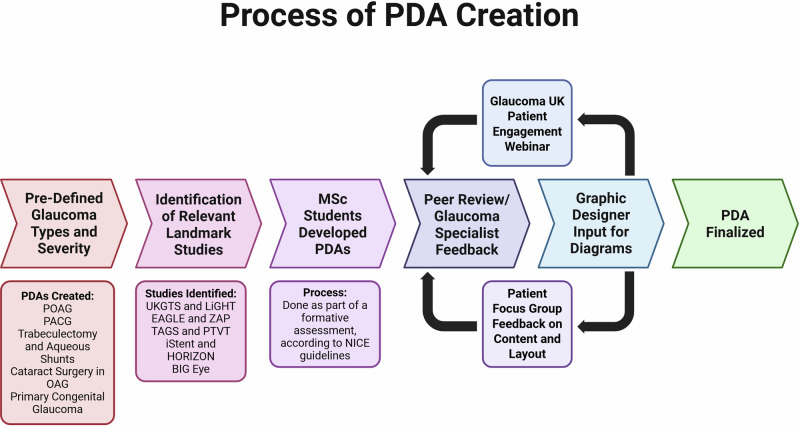
Summary of patient decision aids creation process.

Five key topics were identified for the development of PDAs: mild-to-moderate primary open-angle glaucoma (OAG), advanced OAG, primary angle-closure, cataracts in mild-to-moderate OAG, and primary congenital glaucoma. The selection of these topics was informed by landmark clinical trials [3–11]. 4 PDAs were produced from these topics. Participants for the patient focus group were recruited via a public webinar hosted by Glaucoma UK, which was attended by 67 individuals. Four volunteers took part in the focus group discussion, three of whom participated online, and were compensated with a monetary voucher. The session was transcribed by two clinicians (CH, JKT), and thematic analysis was conducted by CH.

Participants described a lack of consistent communication and conflicting opinions between clinicians, “*Every clinician has their own view on the best way forward*”. Others emphasized the importance of being involved in early treatment decisions. Unclear terminology in the PDA was raised: “*Did not progress… you mean did not get worse?*”.

The accessibility of PDAs was a concern, particularly for those with visual impairments. “*…how else can you communicate this information?*”. There were calls for multi-format versions, including audio and translated versions. Participants also suggested co-designed educational videos: “*… so that they are clear and less ambiguous*”.

Many participants expressed regret at not being fully informed about glaucoma at diagnosis. “*I wish I knew an awful lot more*”. There was desire for the PDA to include an overview of the patient journey: “*Explain what to expect*”.

Difficulties in administering eye drops and remembering complex regimens were commonly cited. “*I had to create my own log to track my drops*”. One participant recalled being given the wrong medication: “*…the packages are so similar*”. Others called for support tools such as drop reminder cards or mobile apps.

Emotional impacts of diagnosis and vision loss were profound. “*You don’t know what you’re missing…*”. Support services such as Glaucoma UK helplines and buddy schemes were described as invaluable. “*So many things I didn’t know…*”. Participants stressed the importance of patients taking an active role: “*It’s teamwork*”.

Patients wanted more information about how glaucoma affects daily life. “*You must reapply for your license at 70. They don’t tell you that*”. Others flagged financial barriers: “*You don’t get free prescriptions until 60*”.

Participants emphasized the need for the PDA to acknowledge and support diverse patient populations. “*Mention higher-risk ethnicities and that family members should get tested*”. Another stressed: “*Not everyone speaks English*”.

Figure 2 summarizes these themes.

**Fig. 2:**
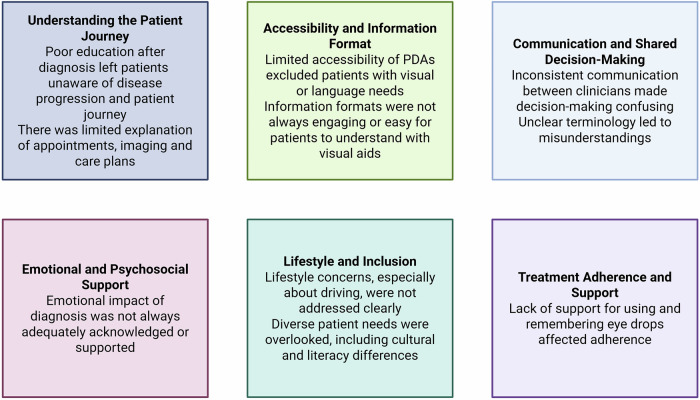
Thematic analysis from patient focus group.

Thematic analysis highlights the importance of glaucoma PDAs being designed to be clear, accessible, supportive, and inclusive. Insights from this focus group offer a valuable framework for enhancing SDM within ophthalmic care. PDAs remain under-utilised in clinical practice, largely due to time constraints during consultations [12]. Their utility is particularly pronounced at initial diagnosis, especially when clinicians have received training in SDM communication techniques. Organizations have a responsibility to ensure that information conforms to the Accessible Information Standard [13]. Landmark trials may not reflect real-world evidence, which can lower efficacy of treatment. Clinicians should therefore tailor the discussions with patients according to local outcomes.

As glaucoma management evolves, PDAs are increasingly integrating risk communication strategies to help patients understand the probabilities of key outcomes associated with various treatment options. These may include the likelihood of achieving intraocular pressure control, preserving vision, or experiencing adverse events. Recent PDAs have also incorporated value clarification exercises, facilitating more meaningful discussions between patients and clinicians. Audio versions of the PDAs are currently in production for visually-impaired patients.

Patients can interact with digital PDAs through decision trees, quizzes, and personalised feedback. However, focus group feedback emphasised the continued need for paper-based versions to ensure accessibility for those who are digitally excluded. In some settings, PDAs have been integrated into electronic health record systems and are provided alongside outpatient letters [14]. Incorporating PDAs into electronic consent processes should be prioritized, although clinicians may be required to aid navigation of the PDA.

Achieving equitable access to PDAs necessitates dedicated funding and resourcing. Targeted efforts are required to ensure that PDAs are accessible to individuals with low vision, limited health literacy, restricted digital literacy, or non-native language proficiency. Furthermore, SDM processes must be culturally sensitive, acknowledging the diverse preferences and backgrounds of patients. To support effective implementation, clinicians may require structured training and system-level support to integrate PDAs seamlessly into clinical workflows [15]. When effectively designed and deployed, PDAs facilitate value clarification, support informed decision-making, and may improve patient engagement, satisfaction, and adherence. Their integration into routine clinical practice represents a critical advancement toward the realisation of patient-centred care.

## Supplementary information

Mild-Moderate Glaucoma Patient Decision Aid

Supplemental File 1 Legend
