# Supplementary material for: Improving patient experience through co-designed patient decision aids in glaucoma
Source: Eye (Lond). 2025 Nov 26;40(1):9–11. doi: 10.1038/s41433-025-04113-5 (PMC12764453; doi:10.1038/s41433-025-04113-5)
Supplement: Supplementary file 1 — Mild-Moderate Glaucoma Patient Decision Aid [file 41433_2025_4113_MOESM1_ESM.pdf]

Patient Decision Aid:

Ocular Hypertension (OHT) and mild to moderate Primary Open Angle Glaucoma (POAG)

This decision aid is designed to support people newly diagnosed with ocular hypertension or open angle glaucoma which is considered mild or moderate.

Section 1: What is ocular hypertension and primary open angle glaucoma?

What is ocular hypertension?

Ocular hypertension is a term which describes any condition where the eye pressure is raised in one or both eyes. The optic nerve (the nerve at the back of the eye) and the field of vision are normal. The drainage pathway appears open.

What are my options for treating ocular hypertension?

If you have ocular hypertension there may be a period of observation before starting treatment. A large study has shown that of those with ocular hypertension who started treatment reduced their chances of developing open angle glaucoma.

Treatment choices include a laser procedure called selective laser trabeculoplasty, or eye drops that lower the pressure in the eye, with the goal of preventing progression to primary open-angle glaucoma.

Treatment choices for ocular hypertension

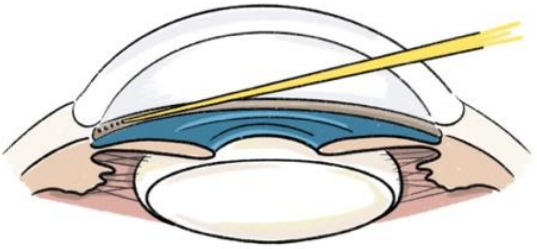

Selective laser trabeculoplasty

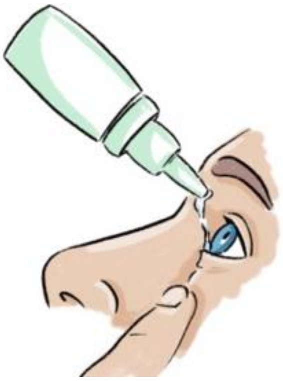

Eye drops

What is primary open-angle glaucoma?

Glaucoma is a condition that affects the optic nerve (the nerve at the back of the eye). In glaucoma the optic nerve becomes damaged over time. This can cause someone's field of vision to be affected. In primary open-angle glaucoma, the drainage pathway in the eye appears open.

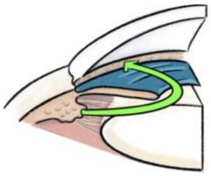

The drainage angle in the eye appears open for aqueous fluid to drain from the eye

→

Despite the open drainage angle, pressure build up within the eye causes damage to the optic nerve

→

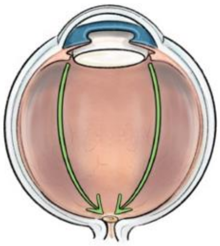

Optic nerve

One of the challenges with glaucoma is that vision loss usually happens very slowly, affecting the very outer parts of the vision first. It does not usually have any other symptoms either. This means many people are unaware that they have glaucoma. The effects of glaucoma on the vision cannot be reversed. It is therefore important that glaucoma is detected early. Reducing the eye pressure is the only way we can treat glaucoma at present. Once you are diagnosed with glaucoma you will need to be under the care of an eye healthcare professional for life.

Damage to the optic nerve, causing loss of outer parts of vision

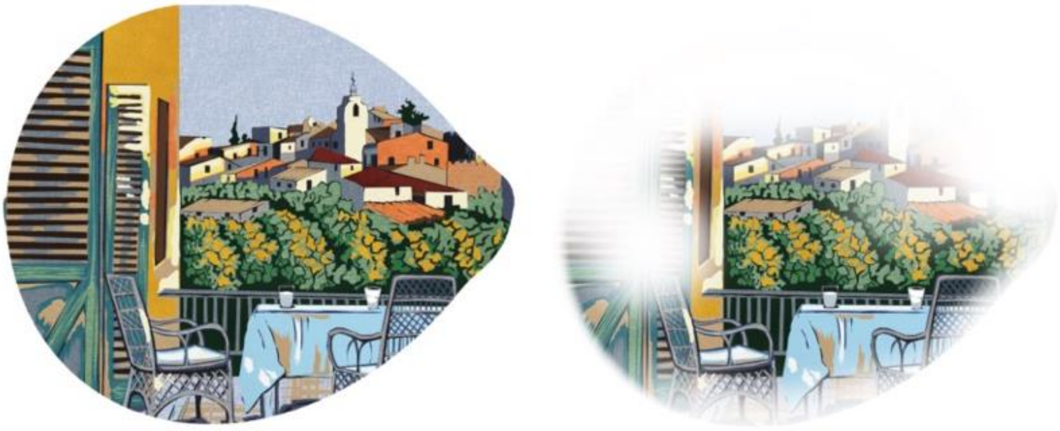

Normal field of vision (one eye)   Damaged field of vision

How common is primary open-angle glaucoma?

Primary open-angle glaucoma is the most common form of glaucoma, and occurs in about 2 in 100 adults over the age of 40 years. Primary open-angle glaucoma is the leading cause of irreversible vision loss worldwide.

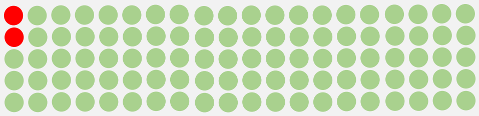

Am I at risk of developing primary open-angle glaucoma?

There are multiple factors that can cause primary open-angle glaucoma.

- A high level of pressure in the eye, known as intraocular pressure, is the most significant factor
- Increased age,
- Having family members that have primary open-angle glaucoma
- Being very short-sighted,
- Some medications e.g. steroids

Section 2: Treatment options for primary open angle glaucoma

What is the goal of treating primary open-angle glaucoma?

The aim of treatment is to slow down further damage to the optic nerve. Reducing the pressure in the eye is the only way we can treat glaucoma at present.

You will need to be monitored closely by your eye healthcare professional, to ensure that your treatment is keeping your eye pressure under control and your field of vision remains stable. It is important to note that the treatment will not cure your glaucoma, but can slow it down.

What are my options for treating primary open-angle glaucoma?

Treatment choices for mild or moderate primary open-angle glaucoma include a laser procedure called selective laser trabeculoplasty, or eye drops that can lower the pressure in the eye.

Both types of treatment aim to lower your eye pressure to slow down worsening of your glaucoma and to prevent it from worsening. A proportion of people may still need to use eye drops after the laser treatment.

Using eyedrops

Once you start eyedrops to lower the pressure in your eyes, you will need to use them every day and roughly at the same time every day **(see link to video on eye drop technique on page 10).**

You will need to ensure that you put in for a repeat prescription in time with your GP and chemist, so you do not run out of eyedrops.

Your eye healthcare professional may ask you to use more than one type of eyedrop in order to reduce your eye pressure sufficiently.

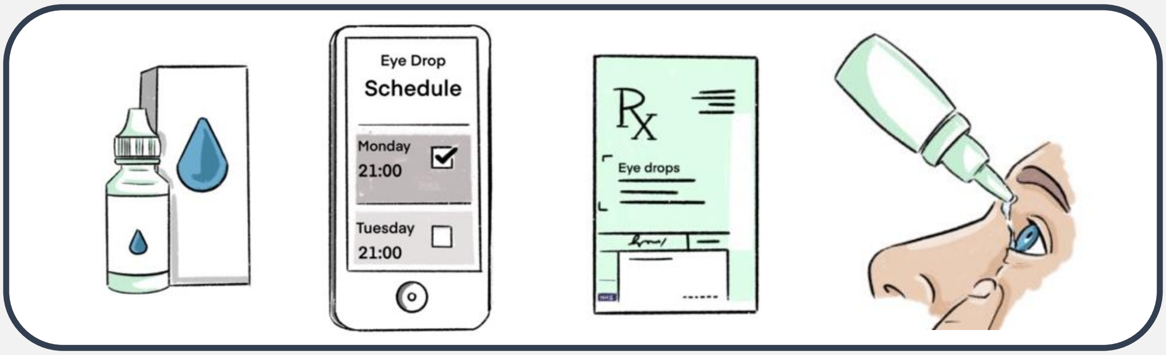

What is selective laser trabeculoplasty?

Selective laser trabeculoplasty involves a laser that uses short pulses of low-energy light and is applied to the eye's drainage system.

This laser procedure improves fluid drainage out of the eye and thereby lowers the pressure in the eye.

The laser procedure is done in the outpatient clinic and takes about 10 – 15 minutes to perform. The laser procedure may be done to both of your eyes during the same visit and you will be expected to wait for a period of time after the procedure to ensure your eye pressure is within the normal expected range.

You may want to bring a relative or friend with you to your appointment as you will be unable to drive home afterwards. The laser usually takes about 6-8 weeks to work.

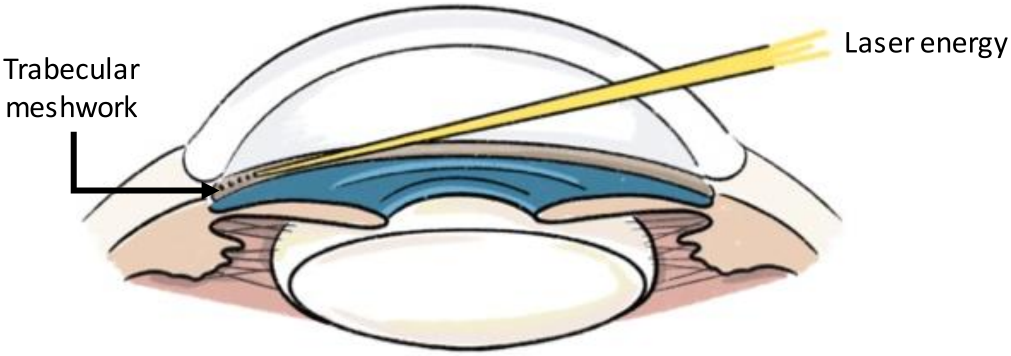

Laser energy is pulsed to the trabecular meshwork (eye's drainage system) to increase drainage of aqueous fluid and reduce pressure build up in the eye

Does selective laser trabeculoplasty hurt?

Anaesthetic drops which numb the surface of the eye will be given before the laser procedure. Most patients report that the laser procedure is fairly painless.

What do I do after the procedure?

The pressure in your eye will be checked after the treatment. You may be given a prescription for a short course of eye drops. A follow-up appointment will be arranged for you to allow eye care professionals to monitor how your eyes have responded to the treatment.

Will I need this procedure again?

It is possible that you will need this procedure again as the effect is not permanent. 20 out of 100 people still need to use regular eye drops to control the pressure in the eye after receiving laser treatment.

What if I don’t want to have this procedure?

If you do not wish to have this procedure, the other option available are eyedrops to lower the eye pressure.

The first eye drop that is usually offered to patients with ocular hypertension or primary open-angle glaucoma is a prostaglandin analogue.

There are other eye drops that can be used in addition. Your eye professional can advise which eye drops are suitable for you.

What if I don’t want to have any treatment for primary open-angle glaucoma?

The decision to not undertake any treatment is your choice, however it is important to bear in mind a few important aspects about this type of glaucoma.

In many patients, primary open-angle glaucoma does not cause any symptoms and so most patients are not aware they have the condition. People with this type of glaucoma only start to notice vision changes and loss in the very advanced stages.

It is also important to note that glaucoma worsens at different rates in different people. For some people it can change very slowly and for others the changes to vision can be very quick.

Therefore, it is important you have regular eye check-ups and decide whether to start treatment together with your eye healthcare professional.

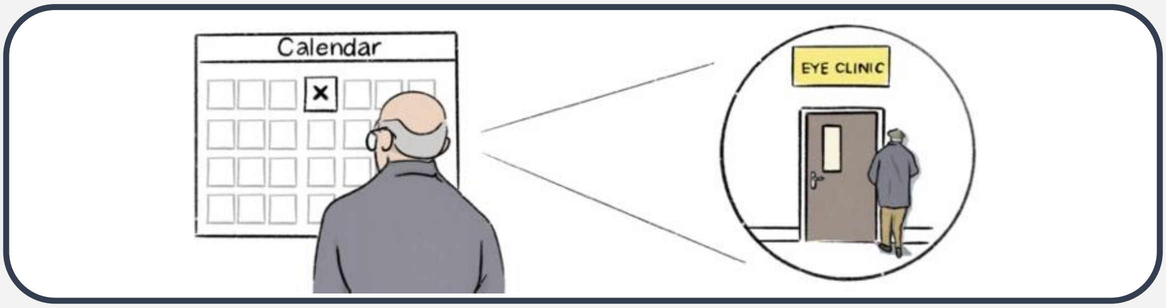

Section 3: Using this decision aid to help you make your choice

Using either of these treatment options will reduce the chances of your condition worsening but these options can impact people differently.

This decision aid is meant to give you information about the advantages and disadvantages of your treatment options, in order to help you and your healthcare professional make the best choice for you.

It is crucial to remember that:

- It is difficult to predict how the vision will change in people with ocular hypertension or primary open-angle glaucoma
- Undergoing treatment for primary open-angle glaucoma will slow or halt damage to the vision in some people, but it may still worsen in others.
- This decision aid is meant for low-risk groups, which can be difficult to accurately identify.

Treatment with eye drops is a long-term, so it is crucial that you have weighed the benefits and costs of your choice. However, you can always change your mind even if your situation changes.

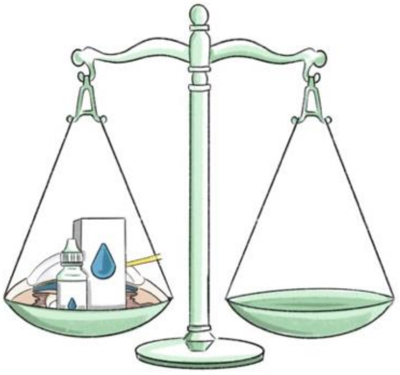

The table at the end of this leaflet is for your own use to help you think about the considerations when starting treatment and how they might impact you and what matters to you.

How important is this to me?

Considerations	Very Important	Important	Unimportant	Very Unimportant
Not using an eye drop every day	☐	☐	☐	☐
Obtaining regular prescriptions for eyedrops and cost of prescriptions	☐	☐	☐	☐
Side effects from prostaglandin analogues eyedrops	☐	☐	☐	☐
Side effects from selective laser trabeculoplasty	☐	☐	☐	☐
How effective treatment is, in reducing my eye pressure or preventing worsening of glaucoma	☐	☐	☐	☐
Maintaining my ability to drive over my life-time	☐	☐	☐	☐
Treatment that is safe in pregnancy and breastfeeding	☐	☐	☐	☐

Section 4: Useful Information

Glaucoma UK

Weblink: https://glaucoma.uk/

- Campaign to raise awareness about glaucoma
- Support people to live well with glaucoma
- Support sight saving research and development
- Advocate with policy-makers, commissioners and care providers.

How to put in your eye drops

Weblink:

https://www.youtube.com/watch?app=desktop&v=G-8NsKIx-sk

Learn more about glaucoma

Weblink:

https://checkout.moorfields.nhs.uk/product?catalog=GLAUCOMA

Things to tell people

- Let your eye doctors know about any change to your health or any new medications – these can impact your eye pressure
- You may want to let blood-relations know about your diagnosis of glaucoma, and encourage them to get their eye test

Section 5: Frequently asked questions

The following pages contain comparison tables detailing observation, medication and surgical or laser intervention for OHT.

The tables are for you to use to help you think about the considerations when starting treatment and how they might impact you and what matters to you.

They aim to answer the following questions:

1. What does this treatment involve?
2. What difference will using this make to my eye pressure?
3. What are common side effects of this?
4. What are the other side effects of this?
5. Will I need to come in for regular tests
6. Will I have to change my lifestyle?
7. Will this interact with any other medication that I am taking?
8. Can I do this if I am pregnant or breastfeeding

What does this involve?

This table is for you to use to help you think about the considerations when starting treatment and how they might impact you and what matters to you.

**Observation**	You will still need to be monitored either by your community optometrist or by a hospital eye service.
**Eye drops**	You will use eye drop once a day in the evening. Treatment is usually long-term.
**Selective Laser Trabeculoplasty**	This involves a laser that uses short pulses of low-energy light and is applied directly to the eye's drainage system.

What difference will using this make to my eye pressure?

Observation

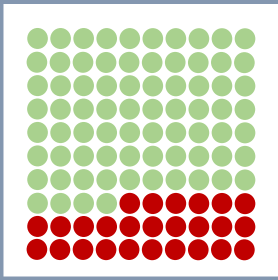

Studies have shown that a large proportion of patients with ocular hypertension and primary open- angle glaucoma will not have any changes to their vision.

74 out of 100 people's glaucoma did **not** worsen after 2 years.

Eye drops

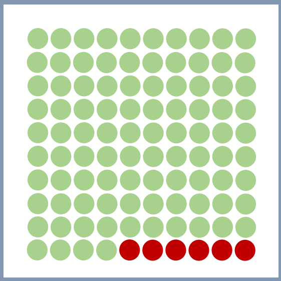

Prostaglandin analogues have been proven to slow worsening of ocular hypertension and mild to moderate primary open-angle glaucoma.

It has also been found to reduce your eye pressure by 3.5 times more than without any eye drops.

94 out of 100 people's glaucoma did not worsen after taking eye drops.

Selective Laser Trabeculoplasty

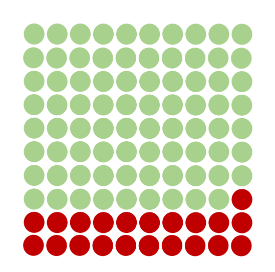

Selective laser trabeculoplasty triggers a change that improves fluid drainage which allows the fluid in the eye to drain more easily, lowering the pressure, reducing the chance of damage to the back of the eye.

79 out of 100 people's glaucoma did **not** worsen after receiving selective laser trabeculoplasty.

What are common side effects of this?

Observation

This is not applicable.

Eye drops

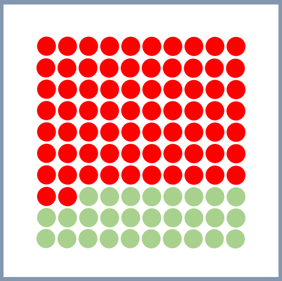

The most common side effects of prostaglandin analogues are thicker and longer eye lashes, darkening of skin around the eye, increased sensitivity to light, irritation and mild redness of your eyes.

72 out of 100 people experienced **mild** side effects from eye drops.

Selective Laser Trabeculoplasty

The most common side effects are blurry vision after the procedure, which will settle after a few hours. You may also have some discomfort and light sensitivity, which usually settles within a week.

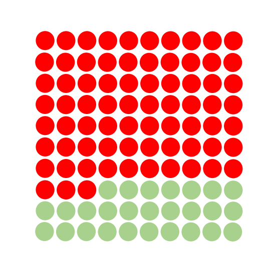

73 out of 100 people experienced **mild** side effects from selective laser trabeculoplasty.

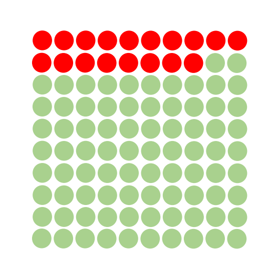

18 out of 100 patients experienced **serious** side effects from selective laser trabeculoplasty.

What are the other side effects of this?

**Observation**	This is not applicable.
**Eye drops**	If you have light-coloured eyes, these can occasionally darken. This is an uncommon side effect. A full list of side-effects can be found in the leaflet found inside the eye drop box.
**Selective Laser Trabeculoplasty**	There is a possibility that the pressure in your eye will rise immediately after this procedure, which will require another surgery to lower. However, this is a very rare occurrence.

Will I need to come in for regular tests

**Observation**	You will still need to be monitored either by your community optometrist or by a hospital eye service.
**Eye drops**	You will be required to come in periodically for routine eye checks to make sure that your eye pressure is stable.<br><br>Your healthcare professional may increase the time between your appointments once your vision and eye pressure have stabilized.
**Selective Laser Trabeculoplasty**	You will be required to attend a follow-up clinic after the procedure to make sure the procedure is having its intended effect.

Will I have to change my lifestyle?

Observation

You will not have make any changes to your lifestyle, although we advise maintaining a healthy diet and active lifestyle.

Eye drops

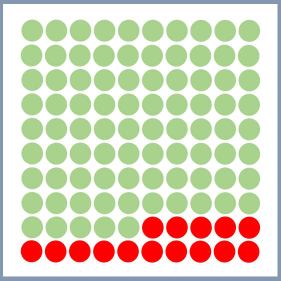

This medication will not require you to make any changes to your lifestyle, although we advise maintaining a healthy diet and active lifestyle.

We advise covering up the inner corner of your eye when using the eye drops to ensure maximum effect.

85 out of 100 patients using eye drops will maintain sight **good enough** to continue to drive (over their lifetime).

Selective Laser Trabeculoplasty

You are advised not to drive yourself to the hospital before this procedure as you will be unable to drive home.

Will this interact with any other medication that I am taking?

**Observation**	This is not applicable.
**Eye drops**	There are no serious common drug interactions with prostaglandin analogues.<br><br>However, you should still speak to your healthcare professional about any medication you are currently taking before starting use of prostaglandin analogues.
**Selective Laser Trabeculoplasty**	No.

Can I do this if I am pregnant or breastfeeding?

**Observation**	Pregnancy can have an effect on your eye pressure due to a change in hormones in your body.<br><br>You should tell your eye healthcare professional if you are pregnant, and they may choose to monitor you more closely.
**Eye drops**	It is recommended to stop using prostaglandin analogues during pregnancy or if you are breastfeeding.<br><br>However, you must let your eye healthcare professional know that you are pregnant, and they will find a suitable alternative treatment for you.
**Selective Laser Trabeculoplasty**	Yes, selective laser trabeculoplasty is safe during pregnancy and breast feeding.
