# Supplementary material for: Improving patient experience through co-designed patient decision aids in glaucoma
Source: Eye (Lond). 2025 Nov 26;40(1):9–11. doi: 10.1038/s41433-025-04113-5 (PMC12764453; doi:10.1038/s41433-025-04113-5)
Supplement: Supplementary file 2 — Supplemental File 1 Legend [file 41433_2025_4113_MOESM2_ESM.docx]

**Supplemental File 1**. Mild-Moderate Glaucoma Patient Decision Aid
